# Identification of markers associated with estimated breeding value and horn colour in Hungarian Grey cattle

**DOI:** 10.5713/ajas.19.0881

**Published:** 2020-05-12

**Authors:** Attila Zsolnai, András Kovács, Endre Kaltenecker, István Anton

**Affiliations:** 1 NAIK-Research Institute for Animal Breeding, Nutrition and Meat Science, Gesztenyés u. 1., 2053 Herceghalom, Hungary; 2 Association of Hungarian Grey Cattle Breeders, Lőportár u. 16.,1134 Budapest, Hungary

**Keywords:** Hungarian Grey, Horn Colour, Estimated Breeding Value, Single Nucleotide Polymorphism (SNP), Genom-Wide Association Study (GWAS)

## Abstract

**Objective:**

This study was conducted to estimate effect of single nucleotide polymorphisms (SNP) on the estimated breeding value of Hungarian Grey (HG) bulls and to find markers associated with horn colour.

**Methods:**

Genotypes 136 HG animals were determined on Geneseek high-density Bovine SNP 150K BeadChip. A multi-locus mixed-model was applied for statistical analyses.

**Results:**

Six SNPs were identified to be associated (−log_10_P>10) with green and white horn. These loci are located on chromosome 1, 3, 9, 18, and 25. Seven loci (on chromosome 1, 3, 6, 9, 10, 28) showed considerable association (−log_10_P>10) with the estimated breeding value.

**Conclusion:**

Analysis provides markers for further research of horn colour and supplies markers to achieve more effective selection work regarding estimated breeding value of HG.

## INTRODUCTION

The phenotypic trits of Hungarian Grey (HG) cattle are characterised by strong pigmentation, long dark eyelashes and well-developed dewlap. The breed was used as a draught animal but it has been bred also for its beef quality. In the 19th century (1884), the majority of the 4.9 million cattle in Hungary were registered as HG. Later at the turn of the century (1900) half of the 6.7 million cattle still belonged to this breed [1]. World War I and II disrupted breeding activity and many herds were destroyed. At the sixties, as a result of the rescue program initiated by Bodó et al [2] and supported by the Hungarian government, cca. 200 purebred cows and six bulls were saved. After its foundation in 1991 the Association of Hungarian Grey Cattle Breeders (AHGCB) remarkably stimulated and coordinated breeding activity. Due to small but permanent subsidies by the state, HG herds started to increase again. Today the HG population amounted to 7,000 cows. More detailed history of the HG are described in several sources [3,4].

Horn of HGs represent a variety in form and in colour. There are white, green and cardy colour varieties where cardy is a mixture of green and white at different ratios [5]. In the first written standard of HGs [6] green horn colour was mentioned as an avoidable trait. Since green colour of horn still persists today as part of the diversity, representing approximately 30% of the population [5], and there are breeders favoring that trait, it is worth to investigate its genetic background.

Nowadays, there is considerable interest in the application of genomic breeding value estimation to promote rapid and efficient selection in farm animals. In the last few years, advances in molecular genetics have enabled the application of DNA-chip technique to pave the way of achieving different breeding objectives. Genom-wide association study based on typing of single nucleotide polymorphisms (SNPs) by DNA chip technique is suitable to find loci associated with economically important traits in different cattle breeds [7], since correlations among genetic background and breeding value of beef can be highlighted by statistical analysis.

At present, breeding decisions in AHGCB are based mainly on maintaining the rotation scheme of the bulls devised to keep diversity at the highest possible level [8]. Inclusion of SNP data affecting intramuscular fat content and meat quality traits into the breeding plan is to be implemented in other breeds e.g. in Hungarian Simmental [9,10] or Holstein-friesian [11].

Estimated breeding values (EBV) signify level of the breeding potential of animals. It is a score of genetic merit combining the relative economic values of several traits. Genetic background of such an EBV evaluating system was investigated in several breeds [12–14]. Such an approach can put the breeding strategy of the breed on new tracks while the original goal of maintaining a gene reserve is to be achieved as well [8].

In this study a genome-wide association analysis has been performed regarding horn colour and EBV (calculation is based on birth weight, daily weight gain, 205 day weight) of HG cattle.

## MATERIALS AND METHODS

### Samples

Samples were collected during routine practice of health investigations organised by the AHGCB previously and independently from the research presented here. Blood samples of 136 HG bulls collected from 16 farms of the country were selected to be genotyped on GeneSeek GGP Bovine 150K SNP chip. Samples have been selected by the AHGCB i) based on their high or low breeding value (ranging from 44 to 188 points), ii) and based on their horn colour.

### Evaluation of estimated breeding value

Evaluation of EBV of HG bulls at 100 to 300 days of age was based on birth weight, daily weight gain, 205 day weight and on maternal data. EBV calculation [15] was based on the following animal model:

y=Xb+Zu+Wm+Spe+e

where y is the EBV, X is the matrix of fixed effects (farm, parturition, date of birth), Z is the matrix of effects of the individual, W is the matrix of maternal genetic effect, S is the maternal permanent environment effect, e is the residual error, b, u, m and pe are the vectors of the corresponding matrices.

### Data evaluation

Series of quality control (QC) procedures were conducted on the raw data [16] using SNP & Variation Suite (SVS) software v.8.8.1 (Golden Helix, Bozeman, MT, USA): monomorphic markers and unmapped SNPs, as well as those with a call rate <95%, were eliminated from the dataset. Duplicated samples (identical by descent value >0.95) and individuals with a genotype call rate <95% were not detected. All samples (n = 136) passed the QC process. After filtering steps, the final dataset contained 126,150 SNPs.

For identification of loci associated with horn colour and EBV, multilocus mixed-models were used [17]. In green vs white horn colour comparisons, the phenotypes (n = 107) were recoded to 0 (white, n = 26) and 1 (green, n = 81). Animals without horn colour data or animals recorded as having cardy horns were discarded from the study. EBV of the animals (n = 136) were recoded to EBV points (EBV_P_) as

$${\text{EBV}}_{\text{P}}=100+20\times [(\text{EBV}-{\text{EBV}}_{\text{MEAN}})/\sigma_{\text{EBV}}]$$

where EBV_MEAN_ is the mean of estimated breeding value, σ_EBV_ is the deviation of EBV.

For the correction of population structure, genomic kinship matrix was used in a multi-locus mixed model [17]. The used model was:

y=Xβ+Zu+e,

where y is the phenotypic value, X is the matrix of fixed effects composed of SNPs and covariates (year of birth and farm), Z is the matrix of random animal effects, e means the residual effects, and β and u are vectors representing coefficients of fixed and random effects, respectively.

## RESULTS

### Association study of horn colour

**Figure 1:**
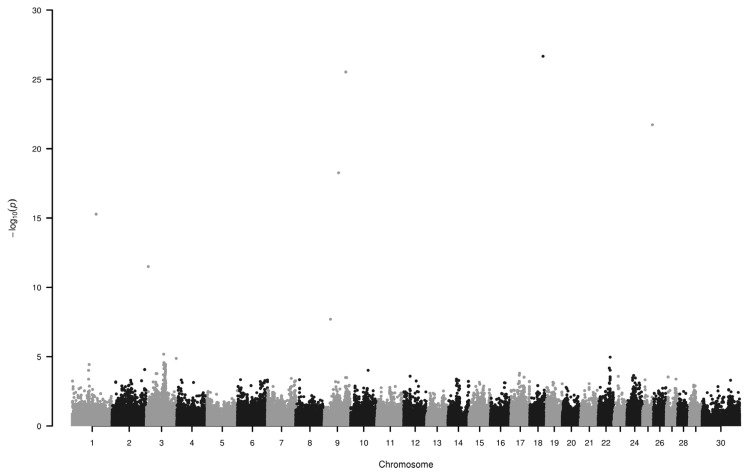
Manhattan plot of single nucleotide polymorphisms associated with green and white horn. Loci on chromosomes 1, 3, 9, 18 and 25 display the highest −log_10_P values. Number 30 on horizontal axis refers to chromosome X.

We have found six loci (−log_10_P>10) on chromosome 1, 3, 9, 18, and 25 to be associated with horn colour (Figure 1; Table 1). Some of the genes around these SNPs (−log_10_P>18) are as follows.

Cromosome 9: Primates and cow EPH receptor A7 were identified as evolutionarily conserved targets of the WNT/beta-catenin signaling pathway [18] and in extracellular region it has two fibronectin type III repeats which can bind to other extracellular matrix proteins [19] such as collagen. Inactivation of uronyl 2-sulfotransferase results altered sulfation of heparan sulfate in mutant neutrophils which affect production of reactive oxygen species and can modulate microbial activity [20]. TGF-beta activated kinase 1 (MAP3K7) binding protein 2 is part of several immune related pathways (https://www.ncbi.nlm.nih.gov/gene/540203) and impairs extracellular matrix homeostasis [21].

On chromosome 18 close to rs110433116 apolipoprotein E has many functions listed in gene ontology (GO) database, including locomotory exploration behavior (GO:0035641), involved in immune processes (GO:0045088, GO:0050728), metal chelating activity (GO:0046911) and also in heparan sulfate proteoglycan binding (GO:0043395).

On cromosome 25, around rs108961742 there are densely packed genes. Three of them, fascin actin-bundling protein 1 (*FSCN1*), actin beta, F-box and leucine rich repeat protein 18 are listed in immune related processes in Panther database. *FSCN1* also plays role in microspike (GO:0030035), microvillus (GO:0032534), and positive regulation of lamellipodium (GO:0010592) assembly.

### Assotiation study of estimated breeding value

**Figure 2:**
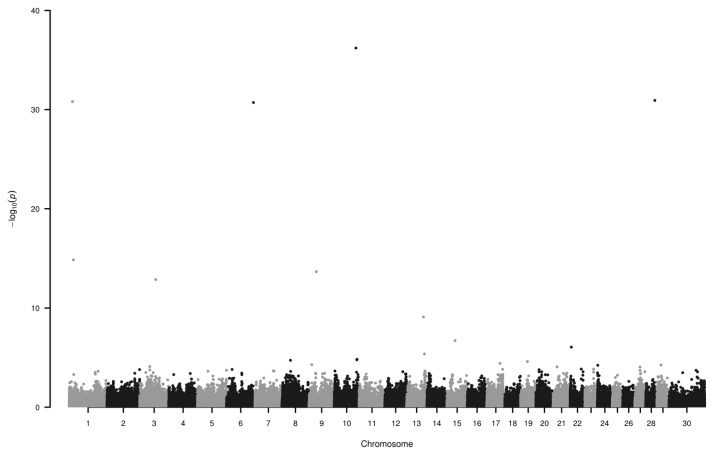
Manhattan plot of single nucleotide polymorphisms associated with estimated breeding value. Loci on chromosomes 1, 3, 6, 9, 10, and 28 display the highest −log_10_P values. Number 30 on horizontal axis refers to chromosome X.

We have found seven loci (−log_10_P>10) associated to EBV on chromosome 1, 3, 6, 9, 10, and 28 (Figure 2; Table 2). Some of the genes around these SNPs (−log10P>13) are as follows.

*Chromosome 1*: Nneural cell adhesion molecule 2 (NCAM2) is similar to immunoglobulin superfamily 4 within their Ig-like C2-type domains, which might play role in tumor supression [22] in humans. In bovine, both NCAM2 and transmembrane serine protease 15 were found to be associated with fat and protein yield [23]. Chondrolectin is predominantly expressed in muscle [24] and its isoform is expressed during T cell maturation [25]. CXADR Ig-like cell adhesion molecule may play critical roles in the response to infection in primary bovine mammary gland epithelial cell [26].

*Chromosome 6*: the actin regulating gene WD repeat domain 1 is downregulated in lactating mammary gland of cattle [27] and its mutation leads to autoinflammatory periodic fever, immunodeficiency, and thrombocytopenia in humans [28]. Heparan sulfate-glucosamine 3-sulfotransferase 1 was found to be associated with eye muscle area in cattle [29]. NK3 homeobox 2 (*NKX3-2*) gene is strongly influencing the fate of cells, e.g. myogenic program of chick muscle satellite cells can be suppressed *in vitro* by chondrogenic factor like *NKX3-2* [30]. It also controls skeletal development [31].

*Chromosome 9*: Serine incorporator 1 is overexpressed in medium follicles of bovine [32]. Heat shock transcription factor 2 is in the upstream region of fatty acid binding protein 4 gene (associated with intramuscular fat content) which might regulate transcription of 3-oxoacyl-(acyl-carrier-protein) synthase 3 protein 4 [33]. Gap junction protein alpha 1 (GJA1) potentially involved in uterine capacity and fertility [34]. According to the GO molecular function terms might play a role in left–right symmetry and embryonic digit morphogenesis (GO:0042733). GJA1 and TBC1 domain family member 32 were also mentioned in association with breeding value of fertility [12]. Minichromosome maintenance 9 homologous recombination repair factor is correlated with estrous behavior in bovine [35]. Anti-silencing function 1A histone chaperone and GJA1 are candidate genes in chiken regarding body composition and meat quality traits [36]. Solute carrier family 35 member F1 is among the predicted target genes associated with marbling score in Korean cattle [37].

*Chromosome 10*: VPS33B interacting protein, apical-basolateral polarity regulator, Spe-39 homolog is associated with infectious hoof lesions in Holstein cattle [38]. It is also among the identified genes involved in mastitis caused by *Escherichia coli* and *Streptococcus uberis* [39]. Global gene expression in the spleen of negative energy balanced dairy cows in the early postpartum period showed, that severe negative energy balance was accociated with immune response, cell death and immunological disease network, where SNW domain containing 1 was upregulated [40]. Iodothyronine deiodinase 2 (DIO2) a thyroid hormone playing critical role in mammalian development and metabolism was found to be associated with body temperature in beef cattle [41]. DIO2 was among the up-regulated genes during primary infection of *Cooperia oncophora* [42]. Thyroid stimulating hormone receptor and neurexin 3 harbor SNPs associated with heifer fertility [43]. They also are associated with puberty in Brangus cattle [44].

*Chromosome 28*: Expression levels of DPY30 domain containing 1 and 2 (DYDC1, DYDC2) and tetraspanin 14 are lower in spermatozoa of high fertility dairy sires [45]. Growth hormone inducible transmembrane protein was found in association with fat-thin tail type in sheep [46]. Coiled-coil serine rich protein 2 is under selection and associated with mastitis in Holstein dairy cows [47].

## DISCUSSION

As for our horn-colour association study, previously Radácsi et al [5] already showed that frequency of the coloured phenotypes did not show differencies among genealogical lines, ages and sexes. The candidate genes, influencing horn colour, around top SNP hits are involved in immune processes, stress pathways, formation of cellular shape, even in metal chelating activity. These genes can serve as a base for devising the upcoming experiments e.g. to investigate algae or bacteria interaction with horn development.

The presented candidate genes around the SNPs associated with EBV are influencing fertility of both sexes, behaviour, body and muscle composition and take part in immune processes.

For the breeders, desiring a shift in green-white colour ratio or wanting to exploit molecular approach in breeding value estimation, the presented markers could be useful in achieving their breeding plans.

## Figures and Tables

**Table 1:** List of loci associated with the green and white colouring of horn, their genomic location and nearest genes

Marker ss ID	Position on chromosomes	−log_10_P	Candidate gene(s) near the marker	MAF	FDR
rs42907907	1: 94860836	15.27	*SPATA16, GPX5, ECT2, GHSR*	0.073	1.3e-11
rs135440681	3: 7761414	11.50	*SH2D1B, ATF6, FCRLB, FCGR2B, HSPA6*	0.378	6.6e-8
rs41593372	9: 57616379	18.26	*EPHA7*	0.427	1.7e-14
rs43602859	9:86470128	25.53	*UST, TAB2*	0.439	1.8e-21
rs110433116	18: 53199067	26.67	*APOE*	0.269	2.7e-22
rs108961742	25: 39404142	21.73	*FSCN1, ACTB, FBXL18*	0.110	7.8e-18

MAF, minor allele frequency; FDR, false discovery rate; *SPATA16*, spermatogenesis associated 16; *GPX5*, glutathione peroxidase 5; *ECT2*, epithelial cell transforming 2; *GHSR*, growth hormone secretagogue receptor; *SH2D1B*, SH2 domain containing 1B; *ATF6*, activating transcription factor 6; *FCRLB*, Fc receptor like B; *FCGR2B*, Fc fragment of IgG receptor IIb; *HSPA6*, heat shock protein family A (Hsp70) member 6; *EPHA7*, EPH receptor A7; *UST*, uronyl 2-sulfotransferase; *TAB2*, TGF-beta activated kinase 1 (MAP3K7) binding protein 2; *APOE*, apolipoprotein E; *FSCN1*, fascin actin-bundling protein 1; *ACTB*, actin beta; *FBXL18*, F-box and leucine rich repeat protein 18.

**Table 2:** List of loci associated with estimated breeding value, their genomic location and nearest genes

Marker ss ID	Position on chromosomes	−log_10_P	Candidate gene(s) near the marker	MAF	FDR
rs132773663	1:13683821	30.81	*NCAM2*	0.006	6.5e-27
rs134031509	1:17714938	14.86	*NCAM2, TMPRSS15, CHODL, CXADR*	0.047	3.5e-11
rs133382330	3:66794402	12.87	*ADGRL4, DNAJB4, MIGA1, USP33*	0.012	2.4e-09
rs135749221	6:112969332	30.71	*WDR1, HS3ST1, NKX3-2*	0.018	6.1e-27
rs109808712	9:30597711	13.66	*SERINC1, HSF2, GJA1, TBC1D32, MAN1A1, MCM9, ASF1A, SLC35F1*	0.012	4.5e-10
rs43651134	10:90679288	36.21	*VIPAS39, SNW1, NRXN3, DIO2, TSHR*	0.012	7.7e-32
rs137560472	28:38224444	30.92	*DYDC1, DYDC2, TSPAN14, GHITM, CCSER2*	0.018	7.5e-27

MAF, minor allele frequency; FDR, false discovery rate; *NCAM2*, neural cell adhesion molecule 2; *TMPRSS15*, transmembrane serine protease 15; *CHODL*, chondrolectin; *CXADR*, CXADR Ig-like cell adhesion molecule; *ADGRL4*, adhesion G protein-coupled receptor L4; *DNAJB4*, DnaJ heat shock protein family (Hsp40) member B4; *MIGA1*, mitoguardin 1; *USP33*, ubiquitin specific peptidase 33; *WDR1*, WD repeat domain 1; *HS3ST1*, heparan sulfate-glucosamine 3-sulfotransferase 1; *NKX3-2*, NK3 homeobox 2; *SERINC1*, serine incorporator 1; *HSF2*, heat shock transcription factor 2; *GJA1*, gap junction protein alpha 1; *TBC1D32*, TBC1 domain family member 32; *MAN1A1*, mannosidase alpha class 1A member 1; *MCM9*, minichromosome maintenance 9 homologous recombination repair factor; *ASF1A*, anti-silencing function 1A histone chaperone; *SLC35F1*, solute carrier family 35 member F1; *VIPAS39*, VPS33B interacting protein, apical-basolateral polarity regulator, Spe-39 homolog; *SNW1*, SNW domain containing 1; *NRXN3*, neurexin 3; *DIO2*, iodothyronine deiodinase 2; *TSHR*, thyroid stimulating hormone receptor; *DYDC1*, DPY30 domain containing 1; *DYDC2*, DPY30 domain containing 2; *TSPAN14*, tetraspanin 14; *GHITM*, growth hormone inducible transmembrane protein; *CCSER2*, coiled-coil serine rich protein 2.
